# Surgical management of endocervical and decidual polyps during pregnancy: systematic review and meta-analysis

**DOI:** 10.1007/s00404-022-06550-z

**Published:** 2022-04-09

**Authors:** Gaetano Riemma, Luigi Della Corte, Salvatore Giovanni Vitale, Stefano Cianci, Marco La Verde, Pierluigi Giampaolino, Luigi Cobellis, Pasquale De Franciscis

**Affiliations:** 1 Department of Woman, Child and General and Specialized Surgery, Obstetrics and Gynecology Unit, University of Campania “Luigi Vanvitelli”, Largo Madonna delle Grazie 1, 80138 Naples, Italy; 2 Department of Neuroscience, Reproductive Sciences and Dentistry, School of Medicine, University of Naples Federico II, Naples, Italy; 3 Obstetrics and Gynecology Unit, Department of General Surgery and Medical Surgical Specialties, University of Catania, Catania, Italy; 4 Dipartimento di Ginecologia Oncologica e Chirurgia Ginecologica Miniinvasiva, Università degli studi di Messina, Policlinico G. Martino, Messina, Italy; 5 Department of Public Health, School of Medicine, University of Naples Federico II, Naples, Italy

**Keywords:** Cervical polyps, Decidual polyps, Pregnancy, Pregnancy loss, Miscarriage, Preterm birth

## Abstract

**Purpose:**

To evaluate the impact of endocervical and decidual polypectomy on obstetrical outcomes of pregnant women.

**Methods:**

MEDLINE, Scopus, ClinicalTrials.gov, Scielo, EMBASE, Cochrane Library at the CENTRAL Register of Controlled Trials, and LILACS were searched from inception to April 2021. No language or geographical restrictions were applied. Inclusion criteria regarded observational studies concerning pregnant women with a cervical lesion who underwent cervical polypectomy. Co-primary outcomes were incidence of late pregnancy loss and preterm birth in women with endocervical or decidual polypectomy as well as polypectomy versus expectant management. Random effect meta-analyses to calculate risk ratio (RR) with 95% confidence interval (CI) were performed. Quality assessment of included papers was performed using Newcastle–Ottawa Scale criteria.

**Results:**

Three studies, with data provided for 3097 women, were included in quantitative analysis, with comparisons between endocervical and decidual polyps extracted from two studies and 156 patients. After a first trimester endocervical or decidual polypectomy, no significant differences were found for late pregnancy losses (RR 0.29 [95% CI 0.05, 1.80], *I*^2^ = 11%). Risk for preterm birth was significantly higher for decidual polyps’ removal (RR 6.13 [95% CI 2.57, 14.59], *I*^2^ = 0%). One paper compared cervical polypectomy vs expectant management, with increased incidence of late pregnancy loss (4/142 vs 5/2799; *p* < 0.001) and preterm birth (19/142 vs 115/2799; *p* < 0.001) in women subjected to polypectomy.

**Conclusions:**

Evidence regarding the removal of cervical polyps in pregnancy is extremely limited. However, the removal of either decidual or endocervical polyps seems associated with increased risk of pregnancy loss and preterm birth, with increased preterm birth risk following endocervical rather than decidual polypectomy.

**Supplementary Information:**

The online version contains supplementary material available at 10.1007/s00404-022-06550-z.

## Introduction

Cervical polyps are a common cause of genital bleeding and vaginal discharge [1]. Their presence is also related to an increased risk of cervicitis and pelvic inflammatory disease due to chronic inflammation [2]. When they are discovered in non-pregnant women, their removal is a feasible way to reduce complaints and abnormal bleeding [3]. It can be done using in-office hysteroscopy without anesthesia with little-to-no discomfort for the patient or by twisting the lesion with forceps [4–8]. Moreover, endocervical lesions should be sent to histopathological analysis for confirming the benignity of the pathology [9–11].

Cervical polyps can be also discovered during pregnancy; they may provoke recurrent bleeding and infection in every phase of the pregnancy, increasing the risk of chorioamnionitis [12, 13]. Moreover, under the influence of pregnancy-related hormones, their size notably increases, with the possibility of a significant protrusion of the lesion toward the external cervical os [14]. For this reason, it can act as a physical obstacle during labor and increase the risk of active bleeding [14].

The role of cervical polypectomy during pregnancy is still debated. It has been reported that the polypectomy during pregnancy significantly reduces the incidence of chorioamnionitis compared to women in which the polypectomy was delayed until delivery [12, 15]. Based on this scenario, some studies recommend the resection of cervical polyps for pregnant women with clinical symptoms (i.e., active genital bleeding or vaginal discharge). However, new evidence shows that cervical polypectomy itself could raise the risk of pregnancy loss and preterm delivery [16, 17].

Moreover, cervical polyps have various histopathological diagnoses. Endocervical polyps, the most common lesions, are hyperplastic protrusions of the endocervical mucosa [18]. During pregnancy, endocervical polyps may undergo a focal stromal pseudodecidualization, which leads the pathologist to commonly diagnose them as “decidual polyps” [2]. It is still unclear whether decidual and endocervical polyps should be treated as a common entity or have different scenarios on the symptoms and on the pursuance of pregnancy.

There are no clear guidelines on whether cervical polyps found during pregnancy should be removed. Subsequently, it currently remains vague whether cervical polyps during pregnancy should be considered a significant harm for late pregnancy loss or spontaneous preterm birth.

To clarify the state of evidence, the aim of this systematic review and meta-analysis was to investigate the impact of endocervical or decidual polypectomy performed during pregnancy on the risk of late pregnancy loss and preterm birth.

## Materials and methods

This meta-analysis was conducted in accordance with the Preferred Reporting Items for Systematic reviews and Meta-Analyses (PRISMA) [19]. The protocol of the systematic review was structured a priori. It outlined strategies for screening the literature, including and examining articles, as well as data extraction, tabulation, integration, and analysis. Therefore, it was registered in the International Prospective Register of Systematic Reviews (PROSPERO) database (CRD42021260847).

### Study search

Seven electronic databases (MEDLINE, Scopus, ClinicalTrials.gov, Scielo, EMBASE, the Cochrane Library at the CENTRAL Register of Controlled Trials, and LILACS) were searched starting from their inception to April 2021. Search terms used were the following text words and Medical Subject Headings (MeSH): “cervical polyps” or “decidual polyps” or “cervical polypectomy” and “pregnancy (MeSH)”. Neither language nor geographic location limitations were adopted. In addition, we screened the reference lists of all eligible papers to retrieve potential studies not captured by electronic searches. The electronic search as well as the eligibility of the selected studies were assessed independently by two authors (G.R. and L.D.C.), while disagreement was solved by involving a third reviewer (P.D.F.).

### Primary and secondary outcomes

Co-primary outcomes of this meta-analysis were the incidence of late pregnancy loss and preterm birth. Late pregnancy loss was defined as a spontaneous interruption of pregnancy occurred after the 12th week of gestational age. Preterm birth was defined as the birth of a living fetus before 37 weeks of gestational age.

### Risk of bias

For studies with an observational design, the risk of bias was judged using the Newcastle–Ottawa Scale (NOS) criteria [20]. According to NOS, each study is evaluated on three broad elements: the selection of study groups, their comparability, as well as the ascertainment of the outcome of interest. Assessment of the selection of a study includes the following criteria: representativeness of the exposed cohort evaluation, non-exposed cohort selection, ascertainment of the exposure of the cohorts, and proof that outcome of interest was not likely to occur spontaneously at the beginning of the study. The comparability of studies is assessed including the evaluation of the comparability of cohorts based on the design or analysis. Moreover, the ascertainment of the outcome of interest is evaluated including the method of determining the outcome of interest, duration, and adequacy of follow-up. According to NOS, a study can be granted a maximum of one star for each numbered item within the Selection and Outcome categories. A maximum of two stars can be awarded for Comparability. Risk-of-bias assessment was independently assessed by three authors (M.L.V., P.G., and S.G.V.). Disagreement was resolved by discussion with a fourth reviewer (P.D.F.).

### Statistical analysis

Two authors conducted the data analysis in an independent manner using Review Manager 5.3 (The Nordic Cochrane Centre 2014, Copenhagen, Denmark) and Stata 14.1 (Stata corp., College Station, TX, 2013).

The summary measures were reported as risk ratios (RR) with 95% of confidence interval (CI) using the random-effects model of Der Simonian and Laird. Higgins I-squared (*I*^2^) index was used to target between-studies heterogeneity. An *I*^2^ index higher than 0% was used to target potential heterogeneity with 25, 50, and 75% identified as cut-offs for low, moderate, and high heterogeneity [21]. The potential publication bias was evaluated by means of the Egger test. A *p* value < 0.05 was considered statistically significant.

## Results

### General characteristics

**Fig. 1:**
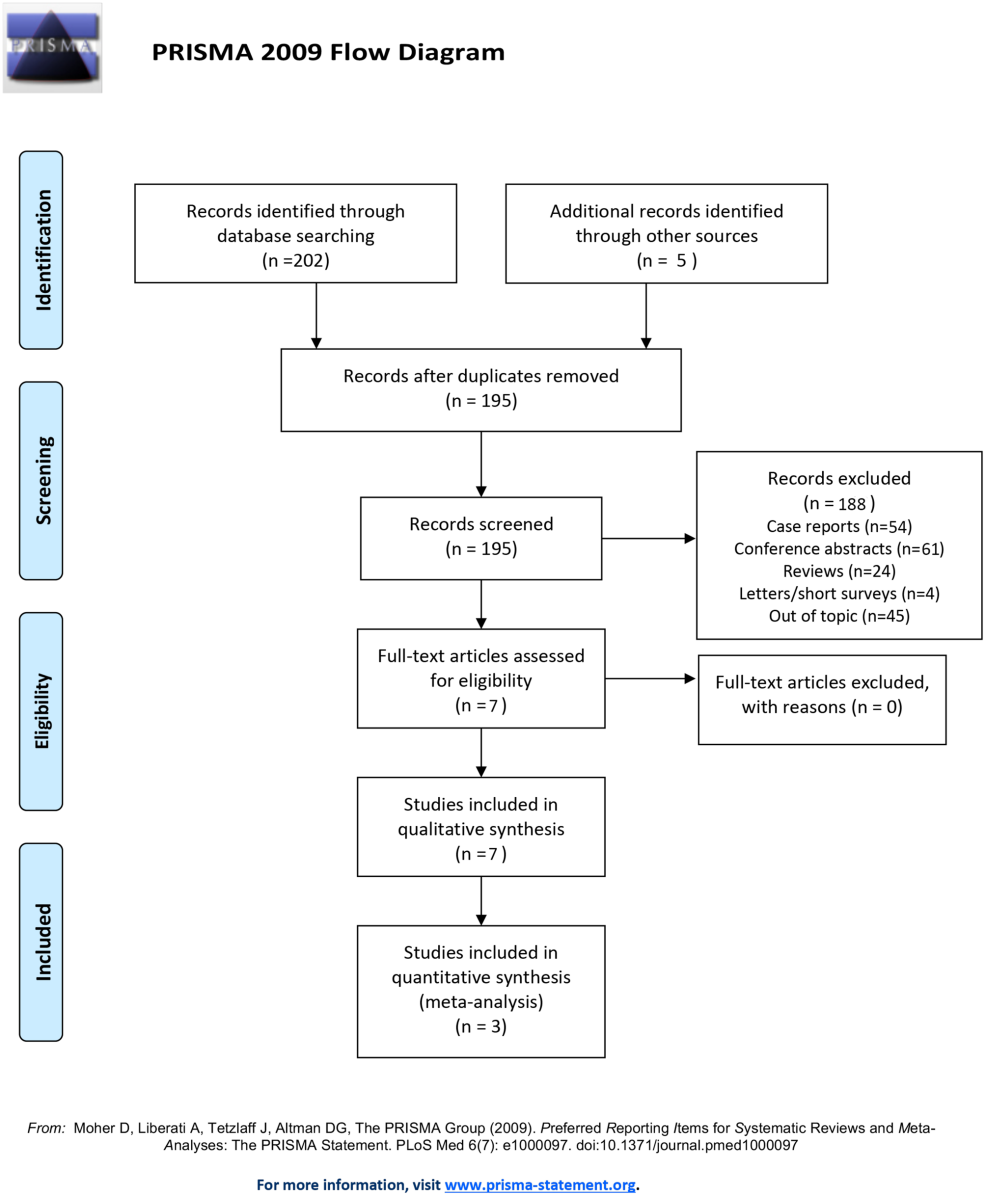
Flowchart of studies included in systematic review and meta-analysis

202 studies were originally identified through database search. Of those, seven were removed as duplicates. After title and abstract screening, 54 studies were removed as out-of-topic case-reports, out-of-topic conference abstracts (61 records) and review articles (24 records), letters/conference papers, or short survey (4 records) or out-of-topic original articles (45 records). Three studies, with data provided for 3097 women, were selected and included in meta-analysis [16, 22, 23] (Fig. 1). Of those, comparisons between endocervical and decidual polyps were retrieved from two studies [22, 23], with data provided for 156 patients. In addition, the above-mentioned studies were supplied by the qualitative analysis of two case-reports [2, 17] and two conference abstracts [24, 25]. Main characteristics of papers included in quantitative synthesis as well as inclusion and exclusion criteria are summarized in Table 1. All the studies were conducted in Japan and had a historical cohort design. One research compared the incidence of late pregnancy losses and preterm birth in women with and without cervical polyp diagnosed in pregnancy [16]. Two studies compared the risk for preterm birth and late pregnancy loss after removal of endocervical lesions in pregnancy [22, 23].

**Table 1:** Baseline characteristics and inclusion/exclusion criteria of studies included in quantitative synthesis

Author, Year	Study design	Study location	Duration	Investigated cohort	Control group	Sample size	Primary outcome	Secondary outcome	Inclusion criteria	Exclusion criteria
Fukuta, 2020	Retrospective cohort	Japan	2003–2017	Women undergoing cervical polypectomy during pregnancy and subsequent PTB or LM	Women undergoing cervical polypectomy during pregnancy and subsequent	73	PTB, LPL, Term delivery	Histopathology of the cervical lesion, Nugent score, incidence of chorioamnionitis	Singleton pregnancies of asymptomatic patients in whom cervical polyps were detected, or women with active genital bleeding from cervical polyps	Pregnant women who delivered because of an obstetric indication (including preeclampsia, placental previa, gestational diabetes mellitus, abruptio placentae)
Hirayama, 2021	Retrospective cohort	Japan	2010–2015	Women diagnosed with in first trimester with a cervical polyp	Women without a cervical polyp during pregnancy	2941	LPL, PTB, Term delivery	PTB before 28 or 34 weeks of gestation	Women with and without a cervical polyp diagnosed in first trimester of pregnancy	Women without a first trimester pregnancy screening
Tokunaka, 2014	Retrospective cohort	Japan	2005–2011	Women undergoing polypectomy for an endocervical polyp	Women undergoing polypectomy for a decidual polyp	83	LPL, PTB	Risk factor for PTB	Pregnant women scheduled to polypectomy of cervical polyps during pregnancy who delivered singleton infants	NR

*LPL* late pregnancy loss, *PTB* preterm birth, *NR* not reported

### Quality characteristics and publication bias

Quality characteristics of included studies, reported by means of the NOS criteria, showed high ratings for all the papers regarding the selection and comparability of the study groups, as well as for ascertainment of the outcomes of interest (Supplementary Table 1).

Publication bias, evaluated by means of the Egger Test, was not apparent (*p* = 0.144).

### Presence of a cervical lesion in first trimester

Hirayama et al. evaluated how the presence of an endocervical lesion (cervical or decidual polyp) might impact on the pregnancy [16]. Even adopting an expectant management, without the removal of the lesion, a cervical organic lesion was associated with poor obstetrical outcomes.

The polyp group showed a statistically significant higher incidence of pregnancy losses (4/142 vs 5/2799; *p* < 0.001) and preterm birth before 28 (3/142 vs 9/3799; *p* = 0.001), 34 (7/142 vs 22/2799; *p* < 0.001), and 37 (19/142 vs 115/2799;* p* < 0.001) weeks of pregnancy.

### Differences between endocervical and decidual polyps

**Fig. 2:**
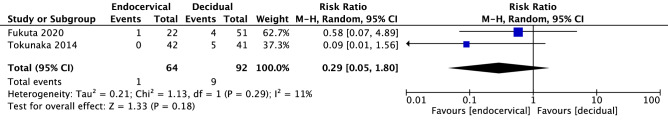
Forest plot for pregnancy loss risk following endocervical vs decidual polypectomy

Two papers evaluated the risk for pregnancy loss after a first trimester endocervical or decidual polypectomy. No significant differences were found between the two groups (RR 0.29 [95% CI 0.05, 1.80], *I*^2^ = 11%) (Fig. 2).

**Fig. 3:**
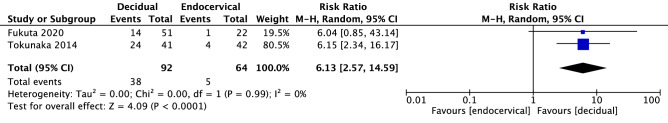
Forest plot for preterm birth risk following endocervical vs decidual polypectomy

The possibility of preterm birth before the 34th week of gestation was analyzed by the same two papers. The removal of a decidual polyp was associated with an increased risk if compared to an endocervical polyp resection (RR 6.13 [95% CI 2.57, 14.59], *I*^2^ = 0%) (Fig. 3).

### Qualitative analysis of case-reports and conference abstracts

Two case-reports described experiences of cervical polypectomies during pregnancy and were included in qualitative analysis. Seo et al. described a single case of first trimester pregnancy loss after a diagnostic polypectomy in a woman with vaginal bleeding [2]. Aoki et al. showed that performing a cervical polypectomy with an Endoloop polydioxanone suture II (Ethicon Endo-Surgery, Germany) led to chorioamnionitis and subsequent premature rupture of membrane at 22 gestational weeks, with spontaneous delivery at 24 weeks and 6 days [17].

The purpose of this review was also discussed by two conference abstracts. Butt et al. showed that, in two primigravidae women, a conservative approach for an endocervical lesion, although with a complaint of first trimester heavy bleeding, is a successful approach to achieve a term delivery, with spontaneous resolution of the polyp after the delivery [24]. Conversely, Yoshida et al. described a case-series of 20 women with a diagnosis of decidual polyp who were managed conservatively. They reported a case of spontaneous pregnancy loss at 13 weeks of gestation, 13 cases of cervical cerclage due to shortened cervical length, and 2 cases of preterm birth before the 28th gestational week, showing the presence of adverse obstetric outcomes even with expectant management [25].

## Discussion

This systematic review found that the presence of an endocervical or decidual polyp is a common risk factor for late pregnancy loss and preterm birth. In case of a cervical polypectomy, neoformations who received a histopathological diagnosis of decidual polyps exhibited an increased risk for preterm birth relative to endocervical mucosal polyps. The risk for late pregnancy loss was the same for decidual and endocervical polyps.

Hirayama et al. gave some insights about the reason for late pregnancy loss in women with and without an endocervical lesion [16]. In their cohorts, four out of five cases of late pregnancy losses in the non-polyp group were due to membrane rupture or fetal membrane prolapse, which all happened after the 20th of pregnancy. Conversely, in the polyp group, premature membrane rupture or acute genital bleeding was diagnosed before the 16th week of gestation, without neither cervical enlargement nor funneling or shortening of the cervix, resulting into earlier pregnancy loss in every patient. Pathological chorioamnionitis was found in all the histopathological samples of those pregnancy losses [14].

Based on this purpose, previous studies report two main causes that can be considered crucial in determining preterm birth. First, cervical polyps become the site of progressive ascending pelvic infection which leads to chorioamnionitis [26, 27]. Second, degeneration and necrosis of cervical polyps cause a considerable release of inflammatory cytokines which are expected to promote cervical ripening [2, 28].

According to that, Kanayama and Terao evaluated both the cervical granulocyte elastase activity and white blood cell count from the cervical mucus of mid-pregnancy women with and without cervical polyps [29]. They reported that both the parameters were significantly higher in women with cervical polyps and were reduced after the polypectomy. In addition, with the histopathological analysis of the placenta, they confirmed an association between cervical polyps and histological chorioamnionitis [29].

An additional concern, as mentioned by Hirayama et al. [16], is that in the majority of women that were conservatively managed, cervical polyps were referred to be spontaneously regressed by the first half of the pregnancy, since they were no longer visible from the external uterine os [29]. However, preterm birth or late pregnancy losses were also found, in their cohort of women, lately during pregnancy [16]. For this reason, it should be emphasized that even if cervical polyps are no longer visible during pregnancy, the risk for spontaneous preterm birth and late pregnancy loss is still present [16, 29]. However, such findings need to be validated by further research.

Moreover, Fukuta et al. emphasized, when the resection of the cervical polyps is unavoidable, to stratify risks in accordance with the size of the lesion, since resected polyps with a diameter of at least 12 mm were significantly related to an increased risk of preterm birth [22].

This systematic review has several limitations. First, the most obvious is related to the extremely low number of papers qualified for meta-analysis. Second, the number of the events investigated, concerning all the outcomes, was relatively small, with one paper providing the vast majority of analyzed women [16]. However, both late pregnancy losses and preterm births are uncommon complications that show low incidence rates also in the general population, as reported by Hirayama et al. in the non-polyp group. An additional limitation is related to the design of available studies, which were all historical cohort analysis, reducing the overall quality of available evidence. Nonetheless, the quality assessment using NOS criteria reported high scores for all evaluated papers, reassuring about the quality of included studies. It should be also acknowledged that, due to ethical constraints, it would be impossible to carry out randomized trials comparing cervical polypectomy to expectant management in pregnant women. Finally, all the studies were carried out in Japan, limiting the possible generalization of the findings to more countries.

Despite the above-mentioned limitations, this review is first quantitative synthesis and meta-analysis on the impact of cervical polyps on late pregnancy losses and preterm delivery.

## Conclusions

Data concerning the removal of cervical polyps during pregnancy are still limited. To date, polypectomy should be avoided on pregnant women, especially in the case of a decidual rather than an endocervical polyp. Suspected malignancy should be considered the only reason for carrying out the procedure. It is still unclear whether this in-office or inpatient surgical treatment is beneficial, and therefore, the operator should use extreme caution when counseling the patient about the risk and benefits, since the risk of spontaneous pregnancy loss or preterm birth may be relevant. Further research should better address the differences between expectant and active management in terms of preterm birth and late pregnancy loss incidence.

## Supplementary Information

Below is the link to the electronic supplementary material.

Supplementary file1 (DOCX 66 KB)

## Data Availability

The data that support this systematic review and meta-analysis are available from the corresponding author, upon reasonable request.

## Declarations

### PROSPERO registration number (date)

CRD42021260847 (July 14, 2021).
