# Supplementary material for: Surgical management of endocervical and decidual polyps during pregnancy: systematic review and meta-analysis
Source: Arch Gynecol Obstet. 2022 Apr 9;307(3):673–80. doi: 10.1007/s00404-022-06550-z (PMC9984338; doi:10.1007/s00404-022-06550-z)
Supplement: Supplementary file 1 — Supplementary file1 (DOCX 66 KB) [file 404_2022_6550_MOESM1_ESM.docx]

**Supplementary Table 1.** Quality scores of the studies included in the meta-analysis, assessed by the Newcastle-Ottawa scale.

|  |  | **Selection** | | | | **Comparability ^a^** | **Outcome** | | | **Overall quality** |
| --- | --- | --- | --- | --- | --- | --- | --- | --- | --- | --- |
| **Author** | **Year** | **Representativeness of the exposed cohort** | **Selection of the non-exposed cohort** | **Ascertainment of exposure** | **Demonstration that the outcome of interest was not present at the start of the study** | **Comparability of cohorts on the basis of the design or analysis** | **Assessment of the outcome** | **Was follow-up enough for outcomes to occur** | **Adequacy of follow-up of cohorts** |  |
| Tokunaka | 2014 | * | * | 0 | * | ** | * | * | 0 | **7** |
| Fukuta | 2020 | * | 0 | * | * | ** | * | * | * | **8** |
| Hirayama | 2021 | * | * | * | * | ** | * | * | * | **9** |

Newcastle-Ottawa scale for assessment of quality of included studies - cohort studies (each asterisk represents if individual criterion within the subsection was fulfilled). ^a^ Comparability of cohorts: for the most important factor: study controls adjusted for gestational age: additional factor: study controls for parity.
